# Supplementary material for: Global warming leads to larger bats with a faster life history pace in the long-lived Bechstein’s bat (Myotis bechsteinii)
Source: Commun Biol. 2022 Jul 9;5:682. doi: 10.1038/s42003-022-03611-6 (PMC9271042; doi:10.1038/s42003-022-03611-6)
Supplement: Supplementary file 2 — Supplementary Information [file 42003_2022_3611_MOESM2_ESM.docx]

## Supplementary information

**Supplements (S) for: Global warming leads to larger bats with a faster life history pace in the long-lived Bechstein’s bat (*Myotis bechsteinii*)**

Carolin Mundinger, Toni Fleischer, Alexander Scheuerlein and Gerald Kerth

## Tables

### Results GAMs

**Table S1:** Summary for the model comparison for **age at first reproduction**. Colony ID and year always entered as random factors. In bold: the best model, selected for its minimal AIC value in combination with smallest degrees of freedom.

| **No** | **Terms** | **df** | **AIC** | **Akaike weights** |
| --- | --- | --- | --- | --- |
| 1 | Intercept | 18.67 | 651.85 | 0.000 |
| 2 | body size | 23.19 | 648.24 | 0.001 |
| 3 | colony size | 23.19 | 643.57 | 0.011 |
| **4** | **body size + colony size** | **21.32** | **634.49** | **0.988** |

**Table S2:** Summary for the model comparison for **fecundity**. Colony ID and year always entered as random factors. In bold: the best model, selected for its minimal AIC value in combination with smallest degrees of freedom.

| **Noo** | **Terms** | **df** | **AIC** | **Akaike weights** |
| --- | --- | --- | --- | --- |
| 1 | Intercept | 8.84 | -14.64 | 0.000 |
| 2 | body size | 6.60 | -31.32 | 0.000 |
| 3 | age at first reproduction | 3.00 | -112.26 | 0.005 |
| 4 | **body size + age at first reproduction** | **6.31** | **-122.92** | **0.995** |

**Table S3a**: Summary for the model comparison for the **costs of reproduction**. Results are given for the full dataset including all years. Colony ID, individual ID and year entered as random factors. In bold: the best model, selected for its minimal AIC value in combination with smallest degrees of freedom.

| **No** | **Terms** | **df**  **all years** | **AIC**  **all years** | **Akaike weights**  **all years** |
| --- | --- | --- | --- | --- |
| 1 | Intercept | 24.06 | 1037.72 | 0.000 |
| 2 | age at first reproduction | 24.53 | 1033.68 | 0.000 |
| 3 | fecundity | 28.93 | 1014.3 | 0.000 |
| 4 | body size | 21.78 | 1032.14 | 0.000 |
| 5 | age | 24.63 | 1010.88 | 0.000 |
| 6 | body size + age | 28.08 | 1010.56 | 0.000 |
| 7 | body size + age at first reproduction | 22.60 | 1028.56 | 0.000 |
| 8 | body size + fecundity | 30.08 | 1017.28 | 0.000 |

**Continuation table S3a:**

| **No** | **Terms** | **df**  **all years** | **AIC**  **all years** | **Akaike weights**  **all years** |
| --- | --- | --- | --- | --- |
| 9 | age at first reproduction + age | 29.55 | 1013.31 | 0.000 |
| 10 | age at first reproduction+ fecundity | 24.87 | 996.33 | 0.000 |
| 11 | age + fecundity | 35.00 | 953.65 | 0.023 |
| 12 | body size + age+ age at first reproduction | 28.08 | 994.56 | 0.000 |
| 13 | body size + age at first reproduction + fecundity | 30.69 | 1016.14 | 0.000 |
| **14** | **age at first reproduction + age + fecundity** | **34.97** | **946.35** | **0.887** |
| 15 | body size + age + fecundity | 36.11 | 956.85 | 0.005 |
| 16 | body size + age + age at first reproduction + fecundity | 38.02 | 951.05 | 0.085 |

**Table 3b:** Summary for the model comparison for the **cost of reproduction (without year 2010)**. Results are given for the dataset without the extreme year of 2010. Colony ID, individual ID and year entered as random factors. In bold: the best model, selected for its minimal AIC value in combination with smallest degrees of freedom.

| **No** | **Terms** | **df**  **without 2010** | **AIC**  **without 2010** | **Akaike weights**  **without 2010** |
| --- | --- | --- | --- | --- |
| 1 | Intercept | 19.56 | 907.49 | 0.000 |
| 2 | age at first reproduction | 20.30 | 900.71 | 0.000 |
| 3 | fecundity | 23.97 | 889.58 | 0.000 |
| 4 | body size | 20.63 | 907.11 | 0.000 |
| 5 | age | 24.39 | 897.54 | 0.000 |
| 6 | body size + age | 26.85 | 895.94 | 0.000 |
| 7 | body size + age at first reproduction | 24.32 | 906.12 | 0.000 |
| 8 | body size + fecundity | 24.95 | 890.8 | 0.000 |
| 9 | age at first reproduction + age | 24.54 | 885.19 | 0.000 |
| 10 | age at first reproduction+ fecundity | 23.91 | 880.51 | 0.000 |
| 11 | age + fecundity | 29.85 | 851.85 | 0.032 |
| 12 | body size + age+ age at first reproduction | 27.17 | 879.54 | 0.000 |
| 13 | body size + age at first reproduction + fecundity | 25.55 | 886.16 | 0.000 |
| **14** | **age at first reproduction + age + fecundity** | **32.78** | **846.02** | **0.580** |
| 15 | body size + age + fecundity | 30.71 | 853.11 | 0.017 |
| 16 | body size + age + age at first reproduction + fecundity | 34.89 | 846.92 | 0.371 |

**Table S4**: Summary for the model comparison for the **lifetime reproductive success** (LRS). Colony ID and birth year entered as random factors. In bold: the best model, selected for its minimal AIC value in combination with smallest degrees of freedom.

| **No** | **Terms** | **df** | **AIC** | **Akaike weights** |
| --- | --- | --- | --- | --- |
| 1 | Intercept | 11.52 | 916.01 | 0.000 |
| 2 | body size | 16.09 | 922.67 | 0.000 |
| 3 | maximum age | 4.50 | 715.16 | 0.000 |
| 4 | age at first reproduction | 13.07 | 919.33 | 0.000 |
| 5 | maximum age + body size | 5.18 | 713.24 | 0.000 |
| 6 | maximum age + age at first reproduction | 5.30 | 698.22 | 0.383 |
| 7 | body size + age at first reproduction | 17.23 | 924.56 | 0.000 |
| **8** | **maximum age + body size + age at first reproduction** | **6.33** | **697.25** | **0.616** |

**Table S5**: Summary for the model comparison for the **likelihood of reproduction** in a given year. Colony ID, individual ID and year always entered as random factors. In bold: the best model, selected for its minimal AIC value in combination with smallest degrees of freedom.

| **No** | **Terms** | **df** | **AIC** | **Akaike weights** |
| --- | --- | --- | --- | --- |
| 1 | Intercept | 21.55 | 2203.30 | 0.000 |
| 2 | body size | 22.61 | 2199.09 | 0.000 |
| 3 | age | 26.2 | 2061.94 | 0.004 |
| 4 | colony size | 22.54 | 2204.33 | 0.000 |
| **5** | **age + body size** | **27.27** | **2051.48** | **0.656** |
| 6 | colony size + size | 23.57 | 2199.65 | 0.000 |
| 7 | colony size + age | 27.24 | 2063.66 | 0.001 |
| 8 | interaction (age + size) | 27.78 | 2077.70 | 0.000 |
| 9  19 | colony size + body size + age | 28.25 | 2052.80 | 0.339 |
| 10 | colony size + interaction (age + size) | 28.74 | 2078.82 | 0.000 |

**Table S6:** Overview of the microsatellite loci used in this study. Provided are the locus name (locus), repeat motif, annealing temperature of the primers (Ta), the multiplex mix that it was included in (Primer mix), fluorescent label (Label), size range of the sequence fragment in base pairs (bp), number of observed alleles, observed heterozygosity (HObs), expected heterozygosity (HExp), polymorphic information content (PIC), GenBank Accession number of the sequenced fragment, and the sequence of the forward and reverse primers used.

* denotes the new primers developed for this study. The other primers are from van Schaik et al. (2018)^32^

| .Locus | **Repeat**  **motif** | **T_a_** | **Primer Mix;**  **Label** | **Size**  **range**  **(bp)** | **No.**  **Alle-les** | **H_Obs_** | **H_Exp_** | **PIC** | **GenBank**  **Accession no.** | **Primer Sequences (5’-3’)** |
| --- | --- | --- | --- | --- | --- | --- | --- | --- | --- | --- |
| F1CYJ | (AAT)11 | 60 | A; FAM | 130-190 | 13 | 0.791 | 0.803 | 0.778 | MG321315 | F:CTTCCTCTTGGTGCTTCCAG  R:GGAGAGCTATCCACCACAGC |
| FLF2N | (AC)18 | 60 | A: PET | 120-170 | 18 | 0.757 | 0.771 | 0.737 | MG321317 | F:GGCTGAGTTTCTTCCAGTGTG  R:TGTAGACATGGCGTGCTAGG |
| FNXJO | (GT)13 | 60 | A; FAM | 220-280 | 21 | 0.868 | 0.861 | 0.845 | MG321319 | F:TTCCACACCTGTGAGCAGAC  RT:GTTTTAACCCACGCAAACACTGAC |
| FQJ6M | (GA)14 | 60 | A; PET | 225-290 | 18 | 0.742 | 0.765 | 0.734 | MG321320 | F:TCAACGGTGCTGCACTAAAC  R:CGGAAGAATTTGGTTCATCTG |
| FYUJN | (CA)17 | 60 | A; VIC | 220-280 | 21 | 0.753 | 0.750 | 0.710 | MG321323 | F:CAGGAAACTGTTGCTGGAGAC  R:GCCTAGTGGAGAATTTATTTGACC |
| GTVIA | (CA)7CG  (CA)10 | 60 | A; NED | 160-220 | 18 | 0.651 | 0.642 | 0.588 | MG321325 | F:ACAGCTGCCAGGAATCTGAC  R:TGACCCAGTCTCCTCCAAAG |
| F4N2G | (AC)13 | 60 | B; PET | 130-170 | 17 | 0.668 | 0.704 | 0.672 | MG321316 | F:CCAGTGCAACTTTGCTAGCTC  R:ACTGCTGATGCCTCTGTTCC |
| FV5AP | (GAT)18 | 60 | B; FAM | 226-265 | 19 | 0.695 | 0.688 | 0.658 | KT013262 | F:AACAGAGTTTGATGGGCTGTTAG  RT:GTTTTGAGGGTGCATGTGTAAGATTC |
| FXB16 | (AC)15 | 60 | B; FAM | 125-210 | 25 | 0.834 | 0.837 | 0.820 | MG321321 | F:AATGGCTGTTTACTGATGAATGG  R:CAGGCTGGGCTGAGAGATAC |
| FZWDE | (AC)13 | 60 | B; HEX | 180-240 | 16 | 0.641 | 0.645 | 0.581 | MG321324 | F:TGAATGCACACAGGCACAC  R:GAGCATGGACCAAGTAAGCAG |
| GD7VI* | (ATT)10 | 60 | B;NED | 165-205 | 15 | 0.699 | 0.697 | 0.658 | OL961308 | F:CTGGAAGGATGCACATAGAGG  R:GCTCAGAGAGGGCACACAG |
| b22 | (GT)10GA(GT)5 | 60 | B; HEX | 120-180 | 16 | 0.747 | 0.766 | 0.739 | MG321313 | F:CTGATGCAAGACCCCTTACAAC  R:ACGGCAGCAGTGAAATCAGA |
| b23 | (CT)24 | 60 | B; NED | 230-290 | 22 | 0.909 | 0.904 | 0.896 | MG321314 | F:CAGTTGGAGGCATGCAGAAA  R:CCGGAGATACTCTTTATTGTTGGT |

**Table S7:** Overview of the form of dataset, response variable, sample size of individuals (N), link function as well all possible predictor variables included in the explanatory GAMs. binom.=binomial

*censored data included: also individuals with unfinished life histories

**included only individuals with finished life histories, who had reproduced at least once during their lifetime

| **Model number** | **Form of data** | **Research question** | **Response**  **variable** | **Smooth**  **function** | **Smooth function** | **Smooth function** | **Fixed factor** | **Random**  **factors** | **Family** | **N Indiv.** |
| --- | --- | --- | --- | --- | --- | --- | --- | --- | --- | --- |
| 1 | summarized over finished lifetime | start of reproduction | age at first reproduction |  | body size | colony size |  | birth year  +  colony ID | Poisson | 225** |
| 2 |  | effect on lifetime fecundity | fecundity |  | body size |  | age at first repro-duction | birth year  +  colony ID | Poisson | 225** |
| 3 |  | effect  on LRS | lifetime reproductive success (LRS) | maximum age mother | body size |  | age at first repro-duction | birth year  +  colony ID | Poisson | 225** |
| 4 | annualized data for every year alive | reproductive rate | reproduction in a given year | age mother | body size | colony size | age at first repro-duction | year  + individual ID  +  colony ID | binom. | 381* |
| 5 |  | mortality rate | death event in a given year | age mother | body size | fecundity rate | age at first repro-duction | year  + individual ID  +  colony ID | binom. | 225** |

## Figures

**Fig. S1:** Boxplots depict the variation in longevity (as age at death) in adult females Bechstein’s bats with different ages of first reproduction.


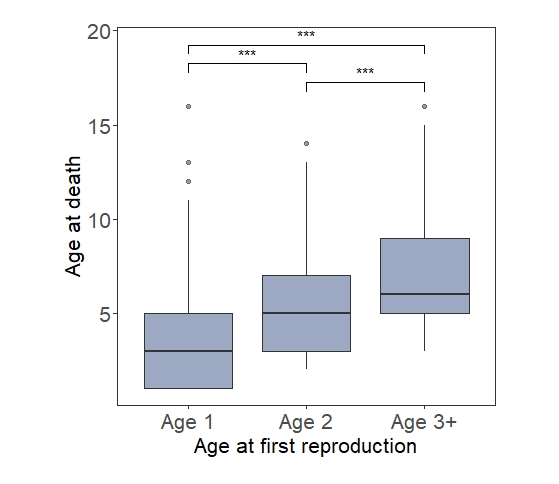


## Supplementary Note 1

We chose a conservative approach including only genetic assignments of the highest confidence. This might lead to an under-estimation of reproductive output, and thus affect especially estimations of LRS, reproductive activity of the different age classes and fecundity rates. For the same Bechstein’s colonies, Fleischer et al. (2017) used observations of lactation status obtained during catching events to estimate breeding rates for different age classes. Although these approaches differed methodically, we found similar patterns in birth rates per age class, albeit our estimates were lower by approximately 10%. Using a subset of our data (1996-2015), that corresponded to the dataset used by Fleischer et al. (2017), we additionally compared LRS estimates calculated based on the 1) genetic matches of the highest confidence, 2) all genetic assignments (regardless of confidence) and 3) lactation observations. Again, we found very similar patterns in LRS between all three methods, with lactation data showing the highest estimates, and the strict genetic assignments the lowest (see Supplementary Fig. 6). This suggest that all three methods lead to comparable results. While our genetic assignment approach might lead to an under-estimation of reproductive output, lactation data might not reflect weaned juveniles accurately, as females will still show sign of lactation although juveniles might have died during earlier stages. This could lead to an overestimation of reproduction. As we focused on successful reproduction events, we deemed the genetic assignments as the better approach.

**Fig. S4**: Overview of LRS estimates, depending on the different methodological approaches. to provide better compatibility, our dataset was subsetted to include the same age classes and years as used in Fleischer et al. (2017). Red line depicts the smooth function for the LRS estimate calculated based using all genetic matches (regardless of confidence level), blue depicts LRS estimate calculated using genetic matches of the highest confidence level (95%), while black depicts LRS estimates based on lactation observation.


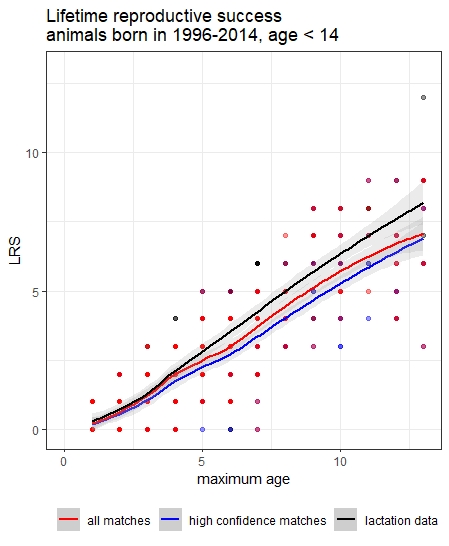

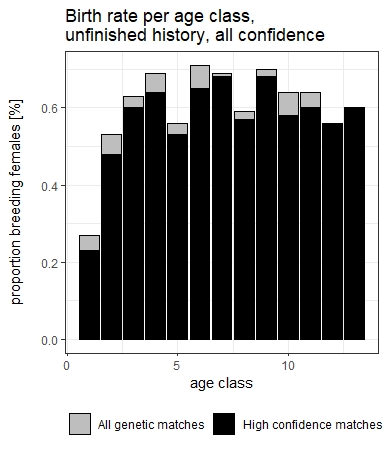


**Fig. S3:** Overview of breeding rates in different age classes and the comparing between the strict approach, only using high confidence assignments, and the relaxed genetic assignments.
