# Supplementary material for: Global warming leads to larger bats with a faster life history pace in the long-lived Bechstein’s bat (Myotis bechsteinii)
Source: Commun Biol. 2022 Jul 9;5:682. doi: 10.1038/s42003-022-03611-6 (PMC9271042; doi:10.1038/s42003-022-03611-6)
Supplement: Supplementary file 3 — Reporting summary [file 42003_2022_3611_MOESM3_ESM.pdf]

nature portfolio

Corresponding author(s): Carolin Mundinger

Last updated by author(s): May 9, 2022

Reporting Summary

Nature Portfolio wishes to improve the reproducibility of the work that we publish. This form provides structure for consistency and transparency in reporting. For further information on Nature Portfolio policies, see our Editorial Policies and the Editorial Policy Checklist.

Statistics

For all statistical analyses, confirm that the following items are present in the figure legend, table legend, main text, or Methods section.

n/a	Confirmed	
☐	☒	The exact sample size (*n*) for each experimental group/condition, given as a discrete number and unit of measurement
☐	☒	A statement on whether measurements were taken from distinct samples or whether the same sample was measured repeatedly
☐	☒	The statistical test(s) used AND whether they are one- or two-sided<br>*Only common tests should be described solely by name; describe more complex techniques in the Methods section.*
☐	☒	A description of all covariates tested
☒	☐	A description of any assumptions or corrections, such as tests of normality and adjustment for multiple comparisons
☐	☒	A full description of the statistical parameters including central tendency (e.g. means) or other basic estimates (e.g. regression coefficient) AND variation (e.g. standard deviation) or associated estimates of uncertainty (e.g. confidence intervals)
☐	☒	For null hypothesis testing, the test statistic (e.g. *F*, *t*, *r*) with confidence intervals, effect sizes, degrees of freedom and *P* value noted<br>*Give P values as exact values whenever suitable.*
☒	☐	For Bayesian analysis, information on the choice of priors and Markov chain Monte Carlo settings
☒	☐	For hierarchical and complex designs, identification of the appropriate level for tests and full reporting of outcomes
☒	☐	Estimates of effect sizes (e.g. Cohen's *d*, Pearson's *r*), indicating how they were calculated

Our web collection on statistics for biologists contains articles on many of the points above.

Software and code

Policy information about availability of computer code

Data collection	No software was used to collect data used in this study.
Data analysis	For the genetic analysis the software GENEMAPPER (V 5.0), CERVUS (V 3.0.7) and Coancestry (V 1.0.1.9) were used.<br>All statistical analyses were performed in R (V 4.1.1). The code is available at Zenodo under doi.org/10.5281/zenodo.5878883

For manuscripts utilizing custom algorithms or software that are central to the research but not yet described in published literature, software must be made available to editors and reviewers. We strongly encourage code deposition in a community repository (e.g. GitHub). See the Nature Portfolio guidelines for submitting code & software for further information.

Data

Policy information about availability of data

All manuscripts must include a data availability statement. This statement should provide the following information, where applicable:

- Accession codes, unique identifiers, or web links for publicly available datasets
- A description of any restrictions on data availability
- For clinical datasets or third party data, please ensure that the statement adheres to our policy

The sequence and primer information for the newly used marker have been submitted to GenBank and are available under ascension no. OL961308.
The datasets analyzed during the current study are available from the corresponding author on reasonable request.

March 2021

Field-specific reporting

Please select the one below that is the best fit for your research. If you are not sure, read the appropriate sections before making your selection.

☐ Life sciences ☐ Behavioural & social sciences ☒ Ecological, evolutionary & environmental sciences

For a reference copy of the document with all sections, see nature.com/documents/nr-reporting-summary-flat.pdf

Ecological, evolutionary & environmental sciences study design

All studies must disclose on these points even when the disclosure is negative.

Study description

We analysed an individual-based 25-year dataset from several hundred RFID-tagged wild bats and combine genetic pedigrees with long-term data on survival, reproduction and body size. We used this data to test whether larger females are able to offset an elevated mortality risk by adopting a faster life history. To do so, we assessed whether size influences reproductive rate and age at first reproduction. We then evaluated the costs of early reproduction on future survival, and investigate the relationship between size and the pace of life. Finally, we compared individual lifetime reproductive success as a measure of fitness across individuals with slow and fast life histories.

In total, 331 females were followed from birth to death, of which 225 individuals reproduced at least once in their lifetime. Including individuals that were still alive at the end of the study period, demographic data were available for a total of 381 females.

Research sample

Research animals are four wild Bechstein's bat (Myotis bechsteinii) colonies living in forests near Würzburg, Germany, with all females individually marked with RFID tags. Bats of the genus Myotis are characterized by long lifespans and low reproductive rates which make them particularly vulnerable to environmental changes and thus highly relevant to study in view of climate change and population persistence.

Only data on female bats was used, as the high natal philopatry of females allows to follow the full life history throughout a completelife time. In contrasts, males leave the colonies to live solitary, so no information on their life history is available.

Sampling strategy

No sample-size calculation was performed. All available data from the last 25 years was used to gain maximum sample sizes.

Data collection

During two capture events each year forearm length was measured to the nearest 0.1 mm using callipers and wing tissue samples were collected for genetic analysis and assignment of maternity. We determined colony size and survival of individuals based on presence-absence data obtained from the annual recaptures and roost monitoring with RFID-readers carried out each year from mid-April to September. Genetic parent-offspring-assignments were used to determine if females had reproduced during a given year, from which we calculated age at first reproduction, lifetime reproductive success and reproductive rates. Data were collected by Prof. Gerald Kerth with the help of various (PhD) students.

Timing and spatial scale

Morphological and demographic data were collected over 25 years, from 1996 to 2020, in four Bechstein's bat colonies. Data on body size and genetic samples were collected annually during two capture events, one in May (after bats have arrived at the summer sites after the hibernation) and one in late July/early August (after birth of juveniles). Roost monitorings with RFID-readers were carried out each year from mid-April to September.

Data exclusions

Mature females alive at the start of the study in 1996 were not considered for the analysis, as data on the complete life history was missing. To analyze trade-offs between reproduction and mortality, we built a discrete time survival analysis framework, including the reproductive parameters 'age at first reproduction' as well as 'fecundity' as further explanatory variables. This required individuals that had complete life-histories (so we could estimate fecundity as the realized lifetime reproduction rate), as well as individuals that had reproduced at least once in their lifetime (so we could include 'age at first reproduction').

We further only included parent-offspring-pairings with the highest confidence matches, which is conservative approach and might lead to an under-estimation of reproductive output, and could thus affect estimations of lifetime reproductive success, reproductive activity of the different age classes and fecundity rates. However, a comparison of methodical approaches (our approach in comparison to parent-offspring-pairings of lesser confidence as well as data on lactation status obtained during catching event) revealed that all three methods lead to comparable results. Lactation data might not reflect weaned juveniles accurately, as females will still show sign of lactation although juveniles might have died during earlier stages. This could lead to an overestimation of reproduction. As we focused on successful reproduction events, we deemed the genetic assignments with the highest confidence levels as the better approach.

Reproducibility

We did not perform any experiments, but recorded the full life history of wild bat colonies. We recorded data from four different colonies and found the same pattern/results across all four colonies, which emphasizes the reproducability of our results. To varify and/or reproduce similar data, a long-term monitoring of wild living bats of sufficient length would be required.

Randomization

Allocation of groups was not random, as Bechstein bats live in closed societies and were thus always a part of the same colony throughout their lifetime. To control for this grouping, we included colony ID as a random factor in the models.

Blinding

Blinding is not relevant for our study, as no experiments were performed and we are not aware of any existing and potential risks of bias.

Did the study involve field work? ☒ Yes ☐ No

Field work, collection and transport

Field conditions	Field work conditions varied between years, but are not relevant to the data analyzed in the study. Nevertheless, to control for varying environmental conditions between years, we included "year" as a random factor in the models.
Location	4 colonies near Würzburg ("Guttenberger Wald", "Unteraltertheim", "Blutsee" and "Höchberg"), Germany
Access & import/export	Habitats are easily accessible, following forest paths by car, then on food. Access permits ("Befahrungsgenehmigungen") for the forests were issued every year by the "Bayerische Staatsforsten". No other samples were imported/exported.
Disturbance	We minimized disturbance by the study by catching bats only outside the sensitive reproduction period and by having trained professionals handling the bats. Additionally, census data throughout the season were collected via non-invasive sampling methods (using automated or hand-held RFID-loggers).

Reporting for specific materials, systems and methods

We require information from authors about some types of materials, experimental systems and methods used in many studies. Here, indicate whether each material, system or method listed is relevant to your study. If you are not sure if a list item applies to your research, read the appropriate section before selecting a response.

Materials & experimental systems

n/a	Involved in the study
☒	☐ Antibodies
☒	☐ Eukaryotic cell lines
☒	☐ Palaeontology and archaeology
☐	☒ Animals and other organisms
☒	☐ Human research participants
☒	☐ Clinical data
☒	☐ Dual use research of concern

Methods

n/a	Involved in the study
☒	☐ ChIP-seq
☒	☐ Flow cytometry
☒	☐ MRI-based neuroimaging

Antibodies

Antibodies used	*Describe all antibodies used in the study; as applicable, provide supplier name, catalog number, clone name, and lot number.*
Validation	*Describe the validation of each primary antibody for the species and application, noting any validation statements on the manufacturer's website, relevant citations, antibody profiles in online databases, or data provided in the manuscript.*

Eukaryotic cell lines

Policy information about cell lines

Cell line source(s)	*State the source of each cell line used.*
Authentication	*Describe the authentication procedures for each cell line used OR declare that none of the cell lines used were authenticated.*
Mycoplasma contamination	*Confirm that all cell lines tested negative for mycoplasma contamination OR describe the results of the testing for mycoplasma contamination OR declare that the cell lines were not tested for mycoplasma contamination.*
Commonly misidentified lines (See ICLAC register)	*Name any commonly misidentified cell lines used in the study and provide a rationale for their use.*

Palaeontology and Archaeology

Specimen provenance	*Provide provenance information for specimens and describe permits that were obtained for the work (including the name of the issuing authority, the date of issue, and any identifying information). Permits should encompass collection and, where applicable, export.*
Specimen deposition	*Indicate where the specimens have been deposited to permit free access by other researchers.*
Dating methods	*If new dates are provided, describe how they were obtained (e.g. collection, storage, sample pretreatment and measurement), where they were obtained (i.e. lab name), the calibration program and the protocol for quality assurance OR state that no new dates are provided.*

☐ Tick this box to confirm that the raw and calibrated dates are available in the paper or in Supplementary Information.

Ethics oversight	*Identify the organization(s) that approved or provided guidance on the study protocol, OR state that no ethical approval or guidance was required and explain why not.*

Note that full information on the approval of the study protocol must also be provided in the manuscript.

Animals and other organisms

Policy information about studies involving animals; ARRIVE guidelines recommended for reporting animal research

Laboratory animals	The study did not involve laboratory animals
Wild animals	We only worked with one species: Bechstein`s bats (Myotis bechsteinii). To obtain measurements and samples of the bats, the bat box containing the bats was carefully taken from the tree during day light. To reduce stress during catching events, bats stayed in these bat boxes with their colony members, until they were carefully extracted from the box to take the necessary measurements and genetic samples. After handling, bats were then directly transferred to a second bat box, where other already handled bats of the same colony were waiting. After finishing the handling, all boxes were hung up on the respective trees they were taken from, so bats were again freely able to leave/enter the box at night.<br>Sample taking during good weather occurred directly in the forest, so no transportation of the bat box was necessary. During rainy weather, bats in their bat boxes were transported to a hut on site (transport of no more than 15 minutes, often less), to prevent the animals from getting wet.<br>In the years 1996-2020 1101 juveniles were registered, 570 female and 531 male juveniles. All adult bats caught were females, which is to be expected, as maternity colonies only consist of adult females. Males live solitary in the forests and do not occupy artificial bat boxes most of the time.
Field-collected samples	Wing tissues samples were stored in 90% ethanol prior to DNA
Ethics oversight	The handling, tagging and monitoring of the bats were conducted under permits for species protection (55.1.-8642.01-2/00) and animal welfare (55.2-DMS 2532-2-20) that had been issued by the government of Lower Franconia.

Note that full information on the approval of the study protocol must also be provided in the manuscript.

Human research participants

Policy information about studies involving human research participants

Population characteristics	*Describe the covariate-relevant population characteristics of the human research participants (e.g. age, gender, genotypic information, past and current diagnosis and treatment categories). If you filled out the behavioural & social sciences study design questions and have nothing to add here, write "See above."*
Recruitment	*Describe how participants were recruited. Outline any potential self-selection bias or other biases that may be present and how these are likely to impact results.*
Ethics oversight	*Identify the organization(s) that approved the study protocol.*

Note that full information on the approval of the study protocol must also be provided in the manuscript.

Clinical data

Policy information about clinical studies

All manuscripts should comply with the ICMJE guidelines for publication of clinical research and a completed CONSORT checklist must be included with all submissions.

Clinical trial registration	*Provide the trial registration number from ClinicalTrials.gov or an equivalent agency.*
Study protocol	*Note where the full trial protocol can be accessed OR if not available, explain why.*
Data collection	*Describe the settings and locales of data collection, noting the time periods of recruitment and data collection.*
Outcomes	*Describe how you pre-defined primary and secondary outcome measures and how you assessed these measures.*

Dual use research of concern

Policy information about dual use research of concern

Hazards

Could the accidental, deliberate or reckless misuse of agents or technologies generated in the work, or the application of information presented in the manuscript, pose a threat to:

No	Yes	
☒	☐	Public health
☒	☐	National security
☒	☐	Crops and/or livestock
☒	☐	Ecosystems
☒	☐	Any other significant area

Experiments of concern

Does the work involve any of these experiments of concern:

No	Yes	
☒	☐	Demonstrate how to render a vaccine ineffective
☒	☐	Confer resistance to therapeutically useful antibiotics or antiviral agents
☒	☐	Enhance the virulence of a pathogen or render a nonpathogen virulent
☒	☐	Increase transmissibility of a pathogen
☒	☐	Alter the host range of a pathogen
☒	☐	Enable evasion of diagnostic/detection modalities
☒	☐	Enable the weaponization of a biological agent or toxin
☒	☐	Any other potentially harmful combination of experiments and agents

ChIP-seq

Data deposition

☐ Confirm that both raw and final processed data have been deposited in a public database such as GEO.

☐ Confirm that you have deposited or provided access to graph files (e.g. BED files) for the called peaks.

Data access links<br>*May remain private before publication.*	*For "Initial submission" or "Revised version" documents, provide reviewer access links. For your "Final submission" document, provide a link to the deposited data.*
Files in database submission	*Provide a list of all files available in the database submission.*
Genome browser session<br>(e.g. UCSC)	*Provide a link to an anonymized genome browser session for "Initial submission" and "Revised version" documents only, to enable peer review. Write "no longer applicable" for "Final submission" documents.*

Methodology

Replicates	*Describe the experimental replicates, specifying number, type and replicate agreement.*
Sequencing depth	*Describe the sequencing depth for each experiment, providing the total number of reads, uniquely mapped reads, length of reads and whether they were paired- or single-end.*
Antibodies	*Describe the antibodies used for the ChIP-seq experiments; as applicable, provide supplier name, catalog number, clone name, and lot number.*
Peak calling parameters	*Specify the command line program and parameters used for read mapping and peak calling, including the ChIP, control and index files used.*
Data quality	*Describe the methods used to ensure data quality in full detail, including how many peaks are at FDR 5% and above 5-fold enrichment.*
Software	*Describe the software used to collect and analyze the ChIP-seq data. For custom code that has been deposited into a community repository, provide accession details.*

Flow Cytometry

Plots

Confirm that:

- [ ] The axis labels state the marker and fluorochrome used (e.g. CD4-FITC).
- [ ] The axis scales are clearly visible. Include numbers along axes only for bottom left plot of group (a 'group' is an analysis of identical markers).
- [ ] All plots are contour plots with outliers or pseudocolor plots.
- [ ] A numerical value for number of cells or percentage (with statistics) is provided.

Methodology

Sample preparation	*Describe the sample preparation, detailing the biological source of the cells and any tissue processing steps used.*
Instrument	*Identify the instrument used for data collection, specifying make and model number.*
Software	*Describe the software used to collect and analyze the flow cytometry data. For custom code that has been deposited into a community repository, provide accession details.*
Cell population abundance	*Describe the abundance of the relevant cell populations within post-sort fractions, providing details on the purity of the samples and how it was determined.*
Gating strategy	*Describe the gating strategy used for all relevant experiments, specifying the preliminary FSC/SSC gates of the starting cell population, indicating where boundaries between "positive" and "negative" staining cell populations are defined.*

- [ ] Tick this box to confirm that a figure exemplifying the gating strategy is provided in the Supplementary Information.

Magnetic resonance imaging

Experimental design

Design type	*Indicate task or resting state; event-related or block design.*
Design specifications	*Specify the number of blocks, trials or experimental units per session and/or subject, and specify the length of each trial or block (if trials are blocked) and interval between trials.*
Behavioral performance measures	*State number and/or type of variables recorded (e.g. correct button press, response time) and what statistics were used to establish that the subjects were performing the task as expected (e.g. mean, range, and/or standard deviation across subjects).*

Acquisition

Imaging type(s)	*Specify: functional, structural, diffusion, perfusion.*
Field strength	*Specify in Tesla*
Sequence & imaging parameters	*Specify the pulse sequence type (gradient echo, spin echo, etc.), imaging type (EPI, spiral, etc.), field of view, matrix size, slice thickness, orientation and TE/TR/flip angle.*
Area of acquisition	*State whether a whole brain scan was used OR define the area of acquisition, describing how the region was determined.*

Diffusion MRI ☐ Used ☐ Not used

Preprocessing

Preprocessing software	*Provide detail on software version and revision number and on specific parameters (model/functions, brain extraction, segmentation, smoothing kernel size, etc.).*
Normalization	*If data were normalized/standardized, describe the approach(es): specify linear or non-linear and define image types used for transformation OR indicate that data were not normalized and explain rationale for lack of normalization.*
Normalization template	*Describe the template used for normalization/transformation, specifying subject space or group standardized space (e.g. original Talairach, MNI305, ICBM152) OR indicate that the data were not normalized.*
Noise and artifact removal	*Describe your procedure(s) for artifact and structured noise removal, specifying motion parameters, tissue signals and physiological signals (heart rate, respiration).*

Volume censoring | *Define your software and/or method and criteria for volume censoring, and state the extent of such censoring.*

Statistical modeling & inference

Model type and settings | *Specify type (mass univariate, multivariate, RSA, predictive, etc.) and describe essential details of the model at the first and second levels (e.g. fixed, random or mixed effects; drift or auto-correlation).*

Effect(s) tested | *Define precise effect in terms of the task or stimulus conditions instead of psychological concepts and indicate whether ANOVA or factorial designs were used.*

Specify type of analysis: ☐ Whole brain ☐ ROI-based ☐ Both

Statistic type for inference (See Eklund et al. 2016) | *Specify voxel-wise or cluster-wise and report all relevant parameters for cluster-wise methods.*

Correction | *Describe the type of correction and how it is obtained for multiple comparisons (e.g. FWE, FDR, permutation or Monte Carlo).*

Models & analysis

n/a	Involved in the study
☐	☐ Functional and/or effective connectivity
☐	☐ Graph analysis
☐	☐ Multivariate modeling or predictive analysis

Functional and/or effective connectivity | *Report the measures of dependence used and the model details (e.g. Pearson correlation, partial correlation, mutual information).*

Graph analysis | *Report the dependent variable and connectivity measure, specifying weighted graph or binarized graph, subject- or group-level, and the global and/or node summaries used (e.g. clustering coefficient, efficiency, etc.).*

Multivariate modeling and predictive analysis | *Specify independent variables, features extraction and dimension reduction, model, training and evaluation metrics.*
